# Variation and Evolution of Human Centromeres: A Field Guide and Perspective

**DOI:** 10.1146/annurev-genet-071719-020519

**Published:** 2021-11-23

**Authors:** Karen H. Miga, Ivan A. Alexandrov

**Affiliations:** 1 UC Santa Cruz Genomics Institute, University of California, Santa Cruz, California 95064, USA; 2 Department of Biomolecular Engineering, University of California, Santa Cruz, California 95064, USA; 3 Department of Genomics and Human Genetics, Vavilov Institute of General Genetics, Russian Academy of Sciences, Moscow 119991, Russia; 4 Center for Algorithmic Biotechnology, Institute of Translational Biomedicine, Saint Petersburg State University, Saint Petersburg 199004, Russia; 5 Research Center of Biotechnology of the Russian Academy of Sciences, Moscow 119071, Russia

**Keywords:** centromere, satellite DNA, repeat, genome, variation, epigenetics

## Abstract

We are entering a new era in genomics where entire centromeric regions are accurately represented in human reference assemblies. Access to these high-resolution maps will enable new surveys of sequence and epigenetic variation in the population and offer new insight into satellite array genomics and centromere function. Here, we focus on the sequence organization and evolution of alpha satellites, which are credited as the genetic and genomic definition of human centromeres due to their interaction with inner kinetochore proteins and their importance in the development of human artificial chromosome assays. We provide an overview of alpha satellite repeat structure and array organization in the context of these high-quality reference data sets; discuss the emergence of variation-based surveys; and provide perspective on the role of this new source of genetic and epigenetic variation in the context of chromosome biology, genome instability, and human disease.

## 1. INTRODUCTION

Centromeres are essential chromosomal structures that mark sites of spindle attachment and ultimately ensure proper chromosome segregation during both mitosis and meiosis. Errors in centromere establishment, inheritance, and maintenance through cell division can result in unequal partitioning of chromosomes and genome instability. Notwithstanding their important cellular function, the precise sequence organization of human centromeres was excluded from initial genome reference assemblies (39, 47, 105) and largely ignored by contemporary genetic and genomic studies over the past two decades. Centromeric regions, and associated pericentromeric heterochromatin, are commonly marked by the enrichment of long arrays of near-identical tandem repeats, or satellite DNAs (91). These highly repetitive sequences have been historically underrepresented due to inherent cloning and sequencing biases: instability in *Escherichia coli* during bacterial artificial chromosome (BAC)-based cloning, the regular occurrence of restriction sites used for cloning in the tandem repeats, or the potential toxicity of the cloned DNA (16, 75). Further, genome assembly methods failed to reliably represent centromeric regions in the past due to the inability to confidently span unique sites in the array that are necessary to predict the linear ordering of thousands of tandem repeats (80, 88). As a result, all human centromeric regions were marked as large gaps, representing megabase-sized placeholders, in our original human reference genomes (39, 47, 105).

Although missing from our initial reference maps, sequences in these regions were not unknown. Focused experimental studies across human centromeric sequences revealed that all normal human centromeric regions are defined at the sequence level by long arrays of alpha satellite DNA, formed by a diverse class of AT-rich tandem DNA repeats, or monomers (58, 59). Individual monomers are commonly arranged into larger, multimonomeric units, or higher-order repeats (HORs) (113), and are organized into one or more highly homogenized arrays at every human centromere. Focused experimental efforts to sample, clone, and directly sequence representative HORs from each centromeric region provided important insight into chromosome-specific subsets of alpha satellite (reviewed in 111), their phylogenetic classification into distinct suprachromosomal families (reviewed in 2), and initial expectations for long-range organization (55, 85, 109, 110). Further, focused studies of the small number of assembled satellites on the chromosome arms adjacent to the centromere gaps of human and nonhuman primate genomes revealed discrete and chronologically ordered alpha satellite layers (81, 85, 89). Emerging databases of alpha satellite–containing reads in whole-genome sequencing data released our first assessments of the frequency of repeat variation within chromosome-assigned arrays, along with early estimates of array length differences between individuals in the population (48, 68, 101). Linear representation of these observed repeat variants and their estimated copy number in the HuRef genome (51) led to the initial release of modeled alpha satellite arrays in the human reference assembly (GRCh38) (68, 104). Although these modeled alpha satellites were inadequate for long-range studies of array structure, they enabled short-read mapping to predict sequence variation (68), detected off-target mapping (66), and offered a more comprehensive study of sequences bound to inner kinetochore proteins (19, 72, 73). Collectively, these extensive studies in the human genome led to the development of the first conceptual representation of centromere genomic organization and sequence evolution across complex genomes.

Advancements in long-read sequencing technologies and recent improvements in repeat assembly methods can now generate complete and accurate assemblies of human centromeric HOR arrays (15, 41, 52, 67, 74). This progress credits the availability of long reads (~15–20 kb) with extremely high consensus base quality (QV30, 99.9%), or high-fidelity (HiFi) sequencing data from Pacific Biosciences (108), and reads that routinely reach hundreds of kilobases in length (40), or ultralong (UL) data from nanopore sequencing from Oxford Nanopore Technologies. In parallel, we have seen tremendous gains in automated satellite array assembly and quality evaluation protocols (15, 69, 74), coupled with standard validation methods using pulsed-field gel electrophoresis (PFGE) and Southern blotting (52, 67).Notably, our current centromeric reference assemblies are derived from an effectively haploid human cell line derived from a complete hydatidiform mole [CHM13hTERT (94)], in which cells have two nearly identical pairs of chromosomes, greatly simplifying the challenge of repeat assembly compared with typical diploid cell lines. The recent release of the first complete assemblies of two human chromosomes end-to-end, or telomere-to-telomere (T2T) [T2T-ChrX (67) and T2T-Chr8 (52)], offered our first opportunity to evaluate these new alpha satellite assemblies in light of the expectations based on previous experimental studies (31, 55, 85) (discussed in more detail later in this review). Further, with the release of additional human centromeric regions (6), we are now met with an opportunity to blend the old with the new: confirming expectations in our original models and highlighting new discoveries with access to complete and accurate maps.

Centromeric satellite repeat copy number and sequence variants within each array are expected to vary considerably (54, 68, 109) due to unequal crossover and conversion. Therefore, a single haploid representation of each human centromeric region is inadequate to comprehensively study the extent of sequence and epigenetic variation. Indeed, satellite repeat copy number estimates across human diversity cohorts, such as the 1000 Genomes Collection (98), have shown that haploid X (DXZ1 or S3CXH1L) and Y (DYZ3 or S4CYH1L) alpha satellite array lengths can differ by a factor of 5–10 between individuals (48, 64, 68) and can be different in two homologous chromosomes from the same person (117). Previous cytogenetic studies have indicated that such variation may contribute to predispositions to cancer, infertility, and chromosomal aneuploidies (28, 116). In addition to centromere sequence diversity, inner kinetochore proteins that bind alpha satellite also show signs of evolving rapidly across primates (86). Further, a scan of the human genome for signatures of positive selection found evidence of recent selective sweeps at 8 of 17 studied centromeres (114), motivating future studies to explore evidence of centromere strength or drive in human population data, as previously documented in other species (10, 25, 45, 46). Ultimately, extensive analysis of the alpha satellite array variation in humans and nonhuman primates will offer new insights into how these regions evolve over time and how such changes influence the localization and inheritance patterns of inner kinetochore proteins.

This is an exciting time for centromere research. Tools are now available to do in-depth analyses of the intersection of genomics and epigenetics to explore variation in centromere structure and models of evolution. Comprehensive studies of the molecular mechanisms that ensure centromere activity will likely provide new insights into human health, and they have the potential to lead to new diagnostic tools and treatments. Additionally, a more complete understanding of centromeres at the genomic level will likely motivate the development of a new era in synthetic genome biology and gene therapy vectors for use in humans. Here, in light of this great progress and promise, we discuss the structure of alpha satellite sequences and our current model of alpha satellite evolution, and we provide a perspective on new studies aimed to improve our understanding of centromere biology and human disease.

## 2. EVOLUTIONARY HIERARCHIES IN ALPHA SATELLITE ARRAY STRUCTURE

### 2.1. Genomic Model of Human Centromeric Regions

Alpha satellite DNAs are credited as the genetic substrate of endogenous centromeres in primates, starting with the new-world monkeys. No alpha satellites have been found in tarsiers and lemurs (49, 89). In humans, arrays of alpha satellites are organized in discrete layers expanding out from a multimegabase-sized homogeneous core that is composed of chromosome-specific HORs (live or active arrays). Additional subsets of alpha satellites (36, 65, 100) are often observed on one or both sides of the core in a near symmetrical formation. This includes a zone of smaller homogeneous HOR arrays (pseudocentromeres or inactive arrays) followed by an outermost layer of progressively more divergent and smaller (center-to-periphery gradient) HOR and monomeric arrays (relic centromeres). Both inactive HOR arrays and divergent arrays are often in the range of a few to hundreds of kilobases. Other distinct satellite classes, such as the classical human satellites (human satellites 1–3, or HSat1–HSat3), are of variable size (up to several megabases) and positioned in the adjacent pericentromeric regions. Segmental duplications are often observed directly flanking the satellite arrays or in centromeric transition regions extending out to the p-arm or q-arm (greater than a megabase) or between adjacent satellite arrays. The entire centromeric region can be defined by those sequences in linkage or sharing a common centromere-spanning haplotype (cenhap), which is characterized by repressed meiotic recombination (48). All alpha satellite arrays except for the active or live HOR arrays may by opposition also be called inactive or dead centromeres or dead centromeric layers.

**Figure 1:**
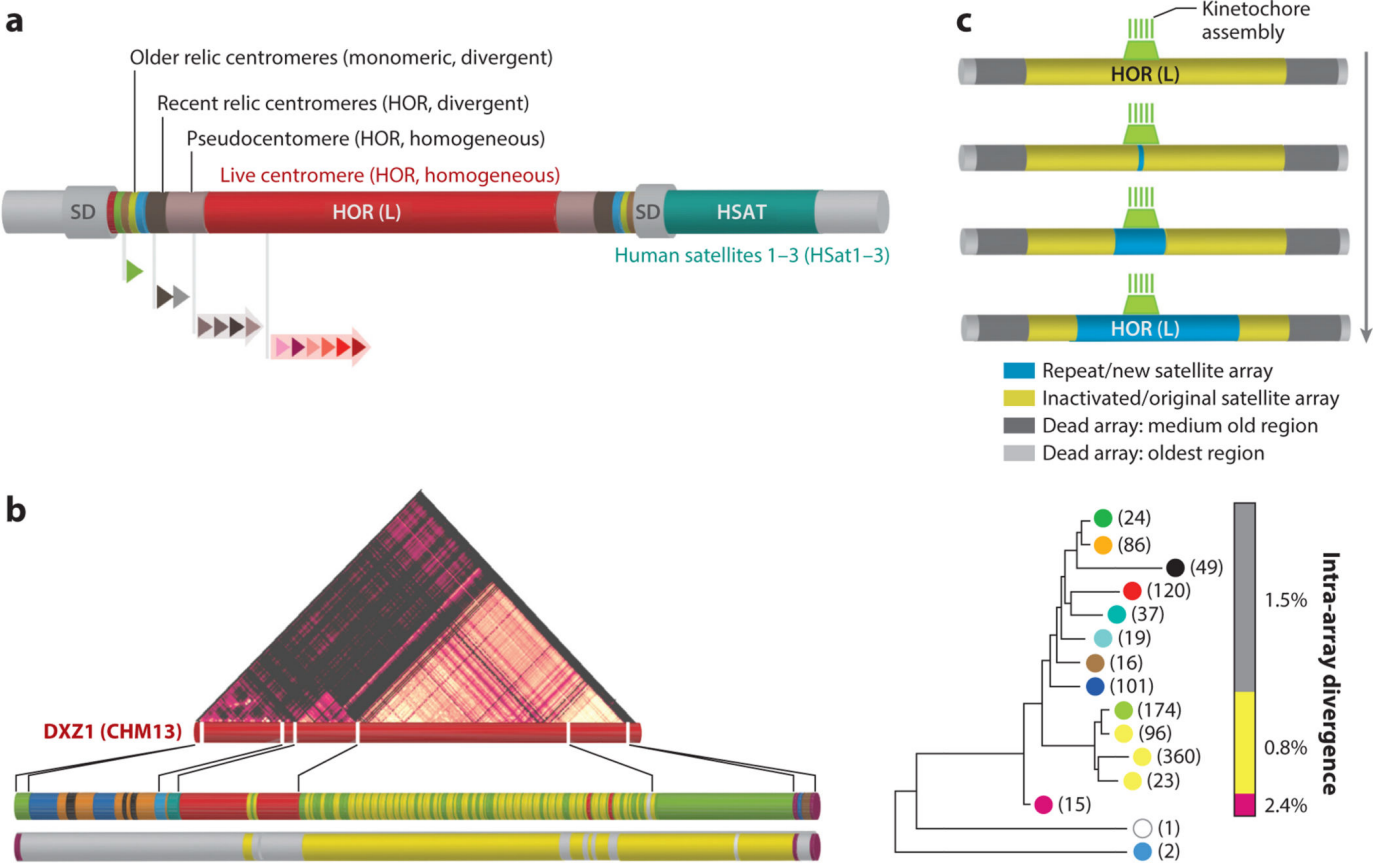
Structure and evolution of alpha satellite arrays. (*a*) Illustration of the general genomic organization of a human centromeric region, which includes one homogeneous core made of chromosome-specific HORs (*red*) and the imperfect symmetrical organization of smaller arrays of various other homogeneous HORs [pseudocentromeres or inactive HOR arrays (*light gray*)], divergent HORs [recent relic centromeres (*dark gray*)], and multiple distinct divergent monomeric arrays (older relic centromeres, with blocks indicating colors describing phylogenetic assignments listed in Supplemental Table 1). These regions typically include other pericentromeric satellite classes [e.g., HSat1–HSat3 (*teal*)] and SDs. The entire centromeric region is defined by those sequences in the cenhap (48), presented as gray flanking regions extending into the p-arm and q-arm. Arrayed triangles indicate alpha satellite monomers and HORs of various length and structures composed of several different monomers. (*b*) Centromere X array haplotype maps, as determined from DXZ1 (S3CXH1L) HOR clustering and divergence data, provide evidence for block organization and gradient of divergence throughout all the layers. Classification of haplotypes is determined by phylogenetic relationships of the DXZ1 HOR repeats, revealing three distinct larger haplotypes (*gray*, *yellow*, and *light purple*). The larger haplotype structure (three major branches on the phylogenetic tree of haplotype consensus HORs) can be further characterized into 14 DXZ1-HOR subgroupings representing individual haplotypes (6, 65). One subbranch (*white*) represented by one HOR is a hybrid between two other haplotypes. The numbers in parentheses indicate the number of HORs in each clade. The dot plot for the self-aligned DXZ1 array (lighter areas have higher homogeneity) and StV map with few variant HORs (*white*) are also shown. (*c*) Kinetochore selection model for satellite array evolution. This model (see Section 2.9) proposes that selfish selection operates on the array through the amplification of the repeat (*light blue*) due to the association with kinetochore (*green*) assembly, which locates itself on repeats to which it happens to have maximal affinity. Over time, the new satellite array (*light blue*) replaces the original satellite array (*yellow*), which shrinks progressively due to the ongoing deletion process. Centromeric arrays that are no longer associated with the kinetochore are considered dead and are arranged symmetrically, flanking the live arrays. Dead arrays are depicted as light gray (oldest region), dark gray (medium old), and adjacent yellow (newly inactivated dead alpha satellite array). Abbreviations: cenhap, centromere-spanning haplotype; HOR, higher-order repeat; HOR (L): live, or HOR array associated with kinetochore assembly; HSat, classical human satellites; SD, segmental duplication; StV, structural variant of a HOR. Figure adapted from data presented in Reference 6.

The general symmetrical disposition of alpha satellite layers around the homogeneous core, which we noted above (see also Figure 1a), reflects the mode of alpha satellite evolution that may be called expanding centromere. It suggests the periodical emergence of a new centromere within the old one (Figure 1c).Analysis of sequence relationships between different HORs within suprachromosomal families (SFs) (see Section 2.4.1) and between dead monomeric layers has shown that centromere expansion likely goes in waves of interchromosomal transfer and amplification, where the HORs (or monomeric sequences) of the newly formed novel centromere jump from one live centromere to another and amplify in the new location to form the next generation of live centromeres (a centromeric layer) in all chromosomes or in a group of chromosomes (2, 4, 85, 89, 112). The remnants of the old centromere are displaced sideways, shrink, diverge, and structurally degrade (see Sections 2.2 to 2.9) (24, 84, 89).

The sequences in the alpha satellite part of a centromere can be characterized by their monomer composition (SF-specific monomer classes; see Section 2.2), their HOR versus monomeric construction, and an average divergence of neighboring copies of a repeat in an array, as there is a gradient of intra-array divergence from the center to the periphery that reflects the age of alpha satellite arrays (2, 42, 84, 85, 89). Also crucial is the functional distinction between active (or live) arrays, which host the kinetochore, and inactive (or dead) arrays, which do not. None of these differences are absolute, and there are many exceptions and borderline cases, but they provide a reasonable way to navigate the centromere landscape. It is also important to note that active centromeres of primates in the human lineage pre-dating the apes were the same in all chromosomes and did not have HORs longer than dimers (panchromosomal organization; the centromere array on chromosome Y is often an exception). Chromosome-specific HORs (i.e., chromosome-specific organization) emerged in the great apes (5, 89). Gibbons are a border case, with evidence for both panchromosomal,or genome-wide, organization and chromosome-specific organization in different taxa (9, 18, 44). Hence, in humans, there are dead relic divergent layers that are the remnants of ancestral primate centromeres (2, 89). Their organization is panchromosomal in older monomeric layers and mostly chromosome-specific in younger divergent HOR layers and even younger homogeneous HOR layers (104).

### 2.2. Alpha Satellite Monomer Classification

Classification of alpha satellite monomers has been summarized in several reviews (2, 36, 63). There are five major alpha satellite SFs. An SF is a group of HOR or monomeric arrays more closely related to each other than to the other groups. Each SF is built of its own set of monomeric classes (see Supplemental Table 1; Section 2.8). The new SFs (SF1–SF3) exist only in African apes (89).They form active (live) centromeres on all human autosomes and the X chromosome (2, 78), most pseudocentromeres (or inactive arrays), and most divergent HORs. The old SF4+ and SF5 unite the dead monomeric layers as well as pseudocentromeres and divergent HOR arrays derived from them by more recent amplifications. SF5 represents centromeres that have been active at the time of the human-orangutan split (89).SF4+ is an umbrella group that unites a large number of old and ancient SFs, such as SF4 proper, SF6, SF7, and more, which correspond to the older primate groups (90). As a notable exception, the active centromere of chromosome Y also belongs to SF4 proper. SF5 is the immediate ancestor of the new SFs (78, 104). It consists of the two monomeric classes R1 and R2, which represent two progenitor types (B and A, respectively) to which all monomeric classes of the new families belong (3, 78). Importantly, the A- and B-type consensus monomers mostly differ from each other in a narrow 17-bp region (the AB-box), which corresponds to the binding site of a well-studied centromeric protein, CENP-B (the B-box, type B monomers) (60), or to the presumed binding site of a very-little-studied (30) pJα protein (the A-box, type A monomers). The relationship of types A and B and SF-specific monomeric classes is shown in Supplemental Table 1.

### 2.3. Archaic Suprachromosomal Families 01 and 02

Recently, Shepelev et al. (90) and Uralsky et al. (104) analyzed a group of the less abundant alpha satellite sequences detected as atypical or archaic representatives of SF1 and SF2. They were shown to be the interim stages of evolution from ancestral SF5 to typical or modern SF1 and SF2. We propose considering them full-fledged SFs, but for the time being, assigning them the incremental numbers 01 and 02, respectively, to avoid renumbering the other SFs. Their monomer classes should be processed in a standard way and included in the SF table (Supplemental Table 1). In the human genome, SF01 and 02 are represented by both divergent (see Section 2.8) and homogeneous HORs, including one live centromere in chromosome 6 (D6Z1 or S01C6H1L), the 3-monomer archaic segment in the live HOR of chromosome 3 (D3Z1 or S01/1C3H1L), relatively large pseudocentromeric arrays in centromeres 3 (S01C3H2) and 20 [S02C20H3; was named HOR20–2 by Shepelev et al. (90)], and also large divergent HOR arrays in chromosomes 3 and 6 [S1C3/6H1d; name changed from S1C3H4 used by Uralsky et al. (104)]. Overall, archaic SF arrays are usually found between the new SFs and SF5 arrays (90). Sequence relationships within SF01 were studied in detail by Uralsky et al. (104).

### 2.4. The Hierarchies in Higher-Order Repeat Domains

Alpha satellite HORs present a complex hierarchy of sequences with different levels of identity between different HORs (coming from different chromosomes or within one chromosome) and different levels of intra-array divergence (2, 4, 6, 15, 36, 63, 65, 81, 90, 104, 111, 112). These levels include SFs, sub-SFs, sister HORs, homogeneous HORs, haplotypes of the same HOR, and divergent HORs, described in the sections that follow.

#### 2.4.1. Suprachromosomal families.

SFs are groups of related HORs that share the same broad classes of monomers (Supplemental Table 1, Section 2.2) and reside on a number of chromosomes (Supplemental Table 2). The divergence between different HORs in one SF is ~12–15% and 20–50% between different SFs (2, 4, 89, 90).

#### 2.4.2. Subsuprachromosomal families.

Sub-SFs are groups of even more closely related HORs within an SF (2, 104).Sub-SFs are known in all new SFs (shown in Supplemental Table 2; e.g., S1C1/5/19H1L, S1C5H2, and S1C16H1L in SF1). Divergence in such groups is ~7–10%.

#### 2.4.3. Sister higher-order repeats.

Sister HORs (Supplemental Table 2) are distinct chromosome-specific sequence variants (major SqVs) within the same HOR that form smaller arrays adjacent to the live HOR [e.g., S3C17H1L (D17Z1), S3C17H1-B (D17Z1-B), and S3C17H1-C (D17Z1-C) (81, 89)] or pseudocentromeric subdomains in the pericentromere [e.g., S3C1H2-A, -B, -C, and -D (104) and S2C18H2-A, -B, -D, and -E (6)]. They are formed by monomers that differ only moderately from respective monomers of the other sister HORs (~3–7%) and may have the same or a somewhat different order of monomers.

#### 2.4.4. Homogeneous higher-order repeats.

Homogeneous HORs (reviewed in 2,36,63) usually have an overall average divergence across the whole array of about 1–2% and are chromosome-specific with a few notable exceptions among the live HOR arrays (double and triple domains) (see Supplemental Table 2; Section 2.5).

#### 2.4.5. Haplotypes of the same higher-order repeat.

Haplotypes of the same HOR (slight SqVs) occupy different regions in the live HOR arrays (6, 15, 52, 65) (see Figure 1b; Section 2.7). A haplotypic HOR region may be formed by one haplotype or by several alternating varieties. Divergence between HORs of different haplotypes may be ~1–3%, and divergence within one haplotype may be as low as 0.5% (see Figure 1b).

#### 2.4.6. Divergent higher-order repeats.

Divergent HORs represent a separate entity that unites HORs that have passed completely or partially through the alleged hypermutability stage and accumulated more divergence than would be possible during their documented or estimated lifespan given the normal mutation rate (see Section 2.8). These are often partially ruined small arrays on the edges of larger homogeneous arrays, some chromosome-specific and some residing on two or several chromosomes. Intra-array divergence is typically over 10% (Figure 1a).

### 2.5. Homogeneous arrays of Higher-Order Repeats

Typical alpha satellite homogeneous HOR arrays consist of chromosome-specific HORs ~4–40 monomers long. However, some nonhomologous pairs of chromosomes share almost identical or very similar live HORs [the so-called paired domains 13/21 and 14/22 and triple domain 1/5/19; reviewed by Alexandrov et al. (2)]. It is not known if these chromosomes have recently shared the centromeres and did not have enough time to diverge or if there is a continuous homogenizing exchange between these chromosomes. This issue could be addressed using the T2T assembly. Some pseudocentromeric HORs are also shared between two or more chromosomes (e.g., S5C5/19H4 is shared by chromosomes 5 and 19; see other examples in Supplemental Table 2).

The traditional naming system for alpha satellite HORs was a part of the more general human gene mapping (HGM) system. It was not very specific or convenient, and many newly discovered HORs did not have HGM names (see discussion in 104). We therefore propose to use the new naming system designed especially for the alpha satellite HORs described by Uralsky et al. (104), which covers all currently known HORs (see proposed names in Supplemental Table 2) and is easier to operate. In this system, each HOR received a name that included its SF, chromosomal location and index number (e.g., S1C13/21H1 for SF1, chromosomes 13 and 21, and HOR#1). Divergent HORs are marked with the d index after the name (e.g., S1C3/6H1d). Live HORs are always H1 and are additionally marked with index L (e.g., S2C9H1L). This new system should be evaluated by satellite and bioinformatic communities to be modified and/or changed as needed. For the time being, we use the old names (whenever they are available) and new names in parallel. Note that no SFs older than SF6 have been found in HORs so far (Supplemental Table 2), new SF1–SF3 (01 and 02 included) are exclusively HOR, and SF5 and SF4 (proper) and SF6 have both HOR and non-HOR arrays (90, 104).

#### 2.5.1. Sequences for CENP-B and pJα binding sites in alpha satellite.

**Figure 2:**
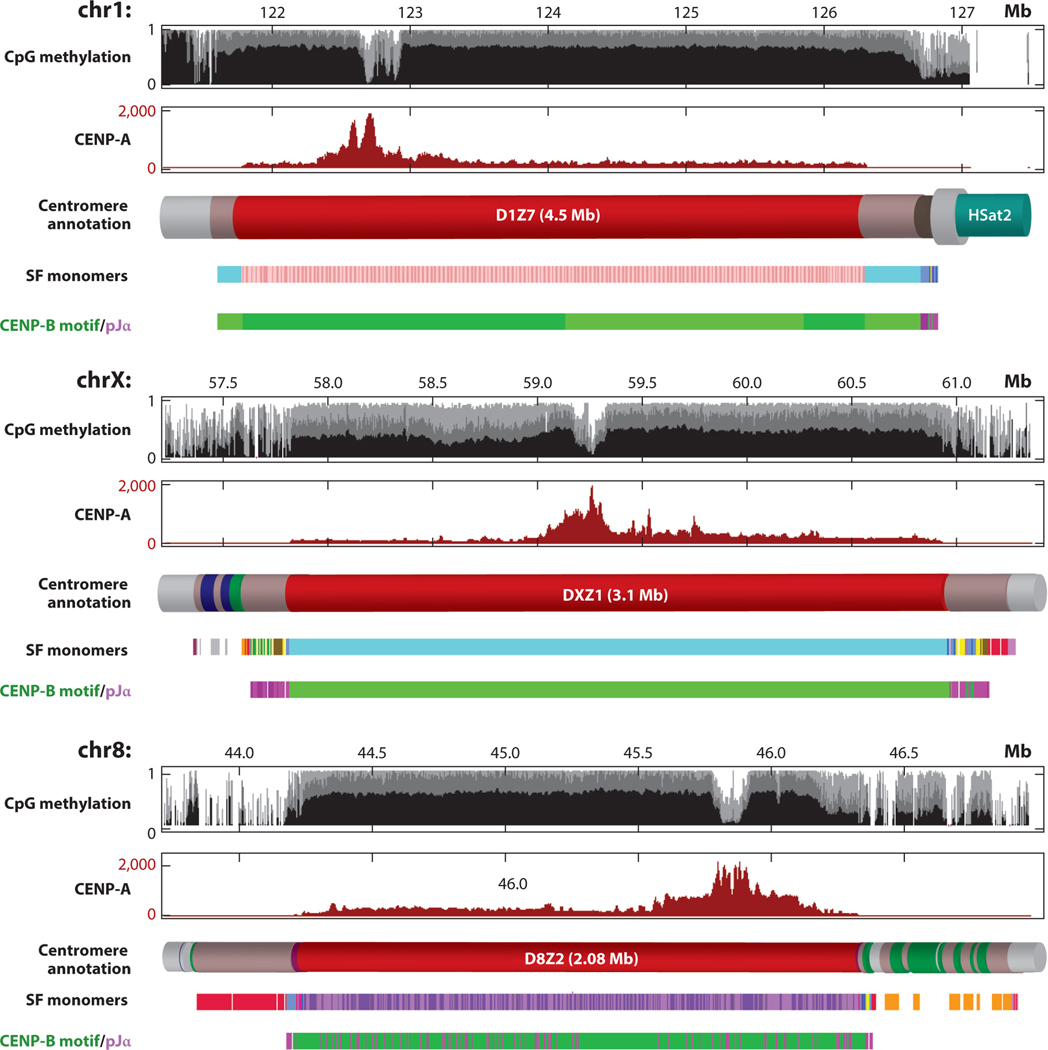
Epigenetic characterization of three complete centromeric arrays from T2T assemblies of chr1, chrX, and chr8. Access to complete and accurate assemblies of human centromeric regions provides a new opportunity to characterize all live alpha satellite HOR arrays [shown for D1Z7, chr1-SF1 (*pink*); DXZ1, chrX-SF3 (*blue*); and D8Z2, chr8-SF2 (*purple*)] and adjunct dead arrays. Further, these maps offer a high-resolution study of CENP-B-binding motifs (*dark green* represents repeats where the motif is in forward orientation and *light green* represents those with a motif in reverse orientation), and pJα-binding site sequences (*light purple*). Note that the regions enriched in reverse motifs indicate an inversion in centromere 1, the single unique event in all of the live centromeres. With the exception of centromere 8 (where CENP-B boxes and pJα are intermixed in the live array), live arrays within centromeric regions on chromosomes 1 and X contain CENP-B boxes, and flanking divergent monomeric regions contain pJα. The map of CpG methylation in ultralong Nanopore data obtained using long-read mapping protocols (previously described in 67) reveals dips in methylation that are coincident with sites of kinetochore assembly [illustrated with enrichment of CENP-A in native ChIP-seq data (52)]. Abbreviations: CENP-A, centromere protein A; CENP-B, centromere protein B; ChIP-seq, chromatin immunoprecipitation sequencing; chr, chromosome; HOR, higher-order repeat; SF, suprachromosomal family; T2T, telomere-to-telomere.

The new SF1–SF3 form all of the live centromeres except for the Y and form most of the pseudocentromeric and relic inactive, or dead, HOR arrays (Supplemental Table 2). In SF1 and SF2 HORs, the J1 and J2 or D1 and D2 class monomers appear as internal J1J2 or D1D2 dimers, respectively, with perfect (SF1) or near-perfect (SF2) AB periodicity across arrays (Supplemental Table 1) (35, 78, 79, 90). In SF3 and SF5, the monomer classes (W1–W5 and R1R2, respectively) alternate in a more complex manner, and the AB pattern also does not have that simple regularity. Note that the presence of the A- or B-box in a monomer does not mean the presence of the actual pJα- or CENP-B-binding site. Boxes are just alternative configurations of the AB region that are permissive to respective sites [35–51 bp of the monomer in our cyclic shift (see 78)]. For this review, we have examined the distribution of the actual sites in the T2T assembly (Figure 2). The actual pJα sites first appear in the Na (*green*) monomers of the dimeric OaNa (*olive-green*) dead layer, but not in the Oa (*olive*) ones (Supplemental Table 1). All of the later successive layers have originated from the green monomer layer only (6, 89), and the sites persist there. In SF5 (R1R2), the B-boxes and actual CENP-B sites first appear, and live centromeres start being formed by the AB satellite (Supplemental Table 1). All new SFs have both the A- and the B-type monomers, but in the human genome, only in SF2 do the pJα sites appear in significant numbers, while CENP-B sites are frequent and regular in all three SFs. Moreover, in SF2, the actual pJα sites are frequent only in some live HORs [e.g., S2C2H1L (D2Z1) and S2C8H1L (D8Z2)], and are virtually absent in many others [e.g., S2C9H1L (D9Z4), S2C14/22H1L, and S2C18H1L (D18Z1)]. Thus, there is possibly an evolutionary trend toward loss of the pJα sites, which may have been in effect since the appearance of the CENP-B sites. If true, it would suggest that both proteins have the same or overlapping functions in centromeres.

#### 2.5.2. Structural variants of a higher-order repeat.

All HORs have structural variants (StVs) that usually can be explained as in/dels of the whole monomers in the primary HOR. Monomer-by-monomer annotation of SF1 reference models in hg38 by Uralsky et al. (104) visualized StVs in HuRef HOR reference models and collected related statistics. Such annotation can now be performed in the T2T CHM13 assembly to collect actual genomic data. Also, the abundant presence of hybrid monomers where a part of one monomer of an HOR was fused to a part of the other was revealed in hg38. The approximate monomer length of ~171 bp is usually conserved in such hybrids. The presence of a hybrid in an StV is a variable feature that depends on a cyclic shift (a monomer start site) used for analysis (15, 21, 22, 104). Therefore, we advocate the use of one universal monomer start site and propose the use of the traditional first nucleotide in the *Bam*HI site of the chromosome X–specific live HOR, which was the first completely sequenced human HOR (106). This cyclic shift was used in about half of the alpha satellite papers over the years and in recently published annotation tools like PERCON, HumAS-HMMER HOR, and CentromereArchitect (22, 90, 104) and is also being used by the T2T consortium for centromere annotation.

Data from Uralsky et al. (104) obtained in hg38 alpha satellite reference models and studies of the first two assembled large centromeres (52, 67) suggest that different live centromeres vary greatly with respect to the abundance of StVs. Some chromosomes, such as X and 11, have non-polymorphic centromeres mainly composed of full-length HOR copies and have only dozens of copies of StVs per 1500–2000 HORs in a centromere. Other centromeres, like 8 and 10, are very polymorphic and have some very high-copy StVs that may exceed the full-length HOR in frequency. It is known that different individual chromosomes (and different persons) may also differ in content and distribution of StVs (1).

### 2.6. Pseudocentromeres and Centromeric Epialleles

Live HOR arrays organize the kinetochore in most individuals, and they are usually the largest HOR arrays in a given chromosome. However, in some individual chromosomes, a smaller, technically pseudocentromeric HOR array may assume the role of kinetochore organizer instead of the main array and form a centromeric epiallele (1, 57; reviewed in 63). We propose that such occasionally functional HORs may be called half-alive or epi arrays, as opposed to the dead ones that are never functional. Then there are two slightly different theoretical possibilities (104): (*a*) half-alive centromeres that had once been live but have surrendered the main centromere status to a more efficient competitor and retained only occasional activity; and (*b*) half-alive pseudocentromeres, which are the HORs that have never been live centromeres but are recent amplifications of some dead alpha satellite sequences that occasionally assume centromeric activity.

### 2.7. Higher-Order Repeat Haplotypes

It has been known for a long time (e.g., 20) that the vast homogeneous core of a centromere formed by nearly identical HORs has some domains made by arrays of even more identical HORs, which share a number of characteristic mutations (a haplotype). Mutations in this case were defined as differences from the overall consensus HOR. Such haplotypes should be considered slight SqVs of a HOR (as opposed to major SqVs, which are sister HORs). Often, they differ not only in sequence but in structure as well, and in those cases they are also StVs. One example of these SqtVs that has been much studied recently is a 13-mer D17Z1 (S3C17H1L) HOR, which is a deleted variant of the complete 16-mer HOR and also differs from it by a number of characteristic mutations (1). In this work, the abundance of this variant HOR in the live arrays of some individual chromosomes 17 apparently prompted the kinetochore to choose an alternative location in the D17Z1-B sister array (1). However, before the complete assemblies of the whole centromeres became available, the data on haplotype patterns within the live arrays were limited. The first two large centromeres assembled by the T2T consortium revealed a considerable heterogeneity of HORs within the live arrays (15, 52, 65). Careful analysis of this heterogeneity (Figure 1b) reveals a phylogenetic tree of haplotypes, a semisymmetric pattern of layers, and a gradient of homogeneity reminiscent of the pattern of pericentromeric dead layers around the live centromere (84, 89). This suggests that the forces and mechanisms operating to create both patterns may partially be the same.

### 2.8. Divergent Alpha Satellite Arrays

Besides live homogeneous centromeres and pseudocentromeres, alpha satellites are found in two kinds of dead (inactive) divergent arrays (HOR and non-HOR), which may be called dead relic centromeres because they represent the actual remains of formerly active centromeres, once large and homogeneous but now small, divergent, and disorderly. These are dead monomeric layers (the remnants of panchromosomal organization of SF4+ and SF5 centromeres) and divergent HOR arrays (the remnants of chromosome-specific SF1–SF3 and SF5 centromeres and pseudocentromeres). SF5 is present in both divisions because it has both HOR and non-HOR components, and some HORs are divergent. In SF4 proper and SF6, both HOR and non-HOR arrays are observed as well, but all HORs found there so far are homogeneous (90). Both divergent compartments share the signatures of a hypermutability phenomenon that has been proposed as a theoretical explanation of their divergence patterns. It has been demonstrated that the intra-array divergence in dead monomeric layers (89) and in divergent HORs (104) far exceeds what could have been accumulated during their lifespan with the normal mutation rate. For instance, in dead monomeric layers of centromere X from Ga [*yellow* (Figure 2; Supplemental Table 1)] to Aa [*gray* (Figure 2; Supplemental Table 1)], the divergence goes from 16% to 30%. Shepelev et al. (89) have speculated that hypermutability in freshly dead arrays is caused by replication problems like fork stalling, which induces error-prone DNA polymerases. The above hypermutability hypothesis is based on the assumption that these arrays were once homogeneous with a divergence not exceeding 1–2%, but a burst of mutations occurred to yield a divergence of >10%. Indeed, there is typically a large gap in intra-array identity between homogeneous (divergence 1–2%) and divergent (>10%) compartments. This could be explained in a traditional way by a special recombination process called homogenization, which is supposed to maintain the large size and high identity in the live arrays. The presumed mechanisms of homogenization are gene conversion (83) and mitotic unequal crossover (92), as meiotic crossover is repressed at centromeres (48, 93). It is obvious, however, that when a new centromere appears in the middle of the old one, as stipulated by the expanding centromere scenario, homogenization stops in the now freshly dead domains, which are displaced to the flanks, and they gradually progress to typical dead centromeres, shrunken, divergent, and disorderly (89). If all of this is true, the time since the centromere has died and homogenization has stopped is the interval in which the array has to accumulate its current intra-array divergence (in excess of ∼2% or less, which it had as a live array). However, it is known that the long-dead arrays accumulate mutations at a normal rate (81, 89). It follows that the accumulation is nonlinear, and the freshly dead arrays must get many more mutations than normal. Shepelev et al. (89) calculated that the excessive divergence gained by freshly dead arrays during the hypermutability period is about 10%, after which the mutation rate subsides. Note that the age of the arrays could be graded by two alpha satellite–dependent (phylogeny of monomers and divergence) and by two alpha satellite–independent ways (89). One of the latter is the presence of the orthologs or paralogs of a given array in extant primate taxa, the age of which is known (34); another is the age of L1 repeats [also known (43)], which often insert into the dead arrays and are very rarely found in the live ones (42).

### 2.9. Conclusions and Evolutionary Models

Sequence mapping shows symmetrical layers of distinct alpha satellite families around and within a homogeneous core, centered at the youngest haplotype(s) in the live array, with the age of layers increasing from the center to periphery (2, 15, 52, 65, 84, 85, 89). Divergence data (Figure 1b; see Sections 2.7 and 2.8) suggest the discontinuous gradient of divergence throughout all the layers, with a minimum at the same youngest haplotype(s) and a steep increase at the transition from homogeneous to divergent compartments (Figure 1a), which can be explained by hypermutability in the freshly dead arrays, presumably caused by induction of error-prone DNA synthesis. Additionally, the degree of structural disorder (a number of deletions, inversions, transposable element (TE) insertions, and HSat expansions) is minimal in homogeneous arrays and much higher in divergent arrays. All of this makes up the signature pattern of an expanding centromere. This pattern suggests a stochastic generation of a new centromeric array inside an existing centromere and lateral displacement of the dead remnants of the old centromere. Through interchromosomal exchange (meant as singular one-way events here), the new repeat spreads to all (or a group of) chromosomes within a short period of time. Such waves of change occur in a regular manner throughout phylogenetic history and create a multilayer centromere, which records its own and its species’ history, similar to archeological layers under a city (89).

Two models may be used to interpret this layout. The neutral homogenization model that dominates so far features stochastic homogenization of neutral mutations, some of which may achieve fixation in all repeats of an array and thus provide for the evolution of an array or the concerted evolution of a number of arrays given sufficient exchange (meant as a continuous two-way process here) between them (92, 95, 96). The next-generation model, which we here term kinetochore selection (Figure 1c), would provide for much faster evolution. This model proposes that (*a*) the evolution of centromeric repeats is not entirely neutral, and they are selected by the affinity to a kinetochore, which is free to move and chooses the most favorable place to reside within the live array; (*b*) this selection operates through the ability of a kinetochore to amplify and possibly homogenize the repeats on which it resides (2, 89); and (*c*) the old centromere abandoned by the kinetochore degrades (deletions, inversions, TE insertions, HSat expansions, and hypermutability). It implies that intense amplification/homogenization is not an intrinsic property of any large array of homogeneous tandem repeats but is dependent on the presence of special machinery, which, in the case of centromeric repeats, is associated with a kinetochore. Hence, the term kinetochore-associated recombination machine or KARM was previously proposed (89). The models are not mutually exclusive, as a mutation or a haplotype favored by a kinetochore probably needs to rise to a certain copy number to compete as a centromere, which may be achieved in a stochastic manner. It also seems that kinetochore selection is entirely compatible with the centromere drive model (56), as both assign a major role to some kind of selfish selection (kinetochore selection or meiotic drive). Presumably, the kinds of selfish selection may be more than one and may be combined easily to better explain the coevolution of centromeric DNA repeats and proteins (56). A somewhat different model for selfish selection in the centromeres was recently proposed by Rice (77). We conclude that the whole process of homogenization has to be rethought as not entirely neutral but as a combination of neutral and selective forces.

## 3. SURVEYS OF GENETIC AND EPIGENETIC VARIATION AT HUMAN CENTROMERES

Centromeric alpha satellite arrays are rich in genetic and epigenetic diversity and present a new and uncharted genomic landscape to catalog structural variation in the human population. Variation in array structure could broaden studies aimed at understanding missing heritability and provide new insight into the genetic basis of complex and rare disorders. Although genome-wide association studies omit centromeric satellite sequences, studies of variants directly adjacent to human centromere arrays, or within centromere-spanning haplotypes, have been observed in a broad number of clinical studies (reviewed in 64), with notable examples in studies of mosaic chromosomal alterations in clonal hematopoiesis (53) and increased risk of multiple sclerosis (76). Efforts to expand our variant maps in centromeric regions are challenging, even with the release of high-quality reference maps, and will require new method development to confidently identify, describe, and test candidate disease causal variants predicted in satellite DNAs. Further, our understanding of disease-associated variants will need to be evaluated in the context of background sampling estimates across the population. We currently do not understand how quickly these sites evolve in the human population, across multigenerational pedigree data, or across a population of single cells. Such fundamental baselines of satellite variation in healthy populations will be critical to confidently identify genetic features associated with disease.

Efforts to measure and report centromere sequencing variation will need to monitor more than the nonamplified mutations that are present in just one copy or few copies. Much of the variation within satellite arrays will be represented as copy number variations, or expansions and contractions of repeat variants, which can give rise to a haplotype (large-scale amplification) or subhaplotype (small-scale amplification). The emergence of more complete T2T genomes will present a new opportunity for method development to predict comprehensive satellite sequence variation by mapping short- and long-read data sets. Previous analyses have demonstrated the use of array assignment of short-read data sets to monitor repeat expansions and contractions through k-mer-assigned frequencies of select satellites (7, 26, 68, 107). These evaluations often report repeat variant information from pooled diploid chromosomes, in which it is not possible to determine copy number variation of the same k-mer present in unequal copies across the two homologous chromosomes without the use of pedigree information or orthogonal phased data sets. High-quality long-read data have been useful in predicting variation in repeat structure (e.g., HOR rearrangements, inversions, transposition, and single-nucleotide variants) as well as in copy number estimates (21, 67, 87). The use of long-read data in satellite DNA variant prediction and discovery is often challenged by inherent biases in sequencing coverage and, in extreme cases, sequencing of only one strand (17, 27), which can influence downstream variant prediction and interpretation.

Ideally, as we reach larger cohorts of completely assembled and properly phased T2T diploid genomes, direct array-to-array comparisons will be possible, allowing direct comparisons of the total length of the array, shared haplotype and subhaplotype repeat expansions, and rare repeat sequences that are not shared between individuals. Such assembly-based comparisons rely on the use of highly accurate sequences to ensure that conclusions are not influenced by introduced assembly error. Ultimately, it is likely that efforts to characterize satellite array variation will need to make a comprehensive assessment of variants to test if features within each array structure (notably, this is a broader definition of a locus, rather than one single-nucleotide polymorphism) are associated with disease.

## 4. NEW PERSPECTIVE ON CENTROMERE GENOMIC STRUCTURE AND FUNCTION

Access to highly accurate reference maps will offer new insight into the range of genomic structure compatible with centromere function. These tools are useful to ensure that confident and precise mapping of short- and long-read functional data sets will promote studies of the positioning of inner centromere proteins and, ultimately, of how variation at the epigenetic level influences chromosome stability during cell division. These emerging mechanistic studies will require a broad, multidimensional view of epigenetics, replication, transcription, and recombination across human centromeric regions.

Although meiotic recombination is suppressed in centromeric regions (12, 55, 62), aligned with the observation of large cenhaps (48), other types of recombination are prolific, leading to repeat amplification, deletions, and inversions. This introduced genetic variation within and between alpha satellite arrays has been shown to influence centromere activity (11, 32). Notably, this has been demonstrated in studies of centromeric epialleles on chromosome 17 (D17Z1 or S3C17H1L; D17Z1B or S3C17H1-B), where HOR size and sequence variants were important features in establishing whether HOR arrays are competent for centromere formation (1, 57). Further, studies of chromosome-specific aneuploidies provide evidence that array composition or particular HOR sequence features [such as the frequency and abundance of CENP-B motifs (61)], rather than the overall array length, influence chromosome segregation fidelity during cell division (19). Studies of the assembled centromeric regions of chromosomes X [DXZ1 or S3CXH1L (65, 67)] and 8 [D8Z2 or S2C8H1L (52)] revealed distinct haplotype blocks where a set of shared HOR variants are localized within the array (52, 67). Careful annotation of entire assembled arrays reveals an uneven distribution of CENP-B motifs across a given array and perhaps indicates collections of repeat units within the array that are less competent for the maintenance of human centromeres (37, 65). Notably, these T2T studies present a snapshot of the precise linear arrangement of HORs within a single individual, and additional studies are critically needed to ensure that we have a more comprehensive understanding of centromere array haplotype blocks within the human population. These data may provide a better genomic context for future centromere genomic studies than general estimates of total array size. That is, the expansion and contraction of variants within specific haplotype arrays provide new insight into segregation fidelity and centromere sequence competency. Indeed, we may find that epialleles exist within a single multimegabase-sized HOR array, and that perhaps the distance between these CENP-A-bound sequences, as shown for other dicentric chromosome models (97, 99), may contribute to our understanding of centromere strength (46).

Focusing exclusively on the enrichment patterns of inner kinetochore proteins with the underlying alpha satellite DNAs may provide an incomplete picture of the epigenetic determinants of centromere identity and function. Kinetochore assembly is observed over a small proportion of the array, with flanking regions enriched in pericentric heterochromatin and CpG methylation (29, 82). The dynamics between centrochromatin domains (13) and pericentromeric heterochromatin may have broad influence, from spatial localization in the interphase nucleus (8), the formation of three-dimensional structure during mitosis (13), and low transcription and increase in chromosomal passenger complex occupancy (71). Therefore, the use of the new alpha satellite assemblies will provide a unique opportunity for the comprehensive analysis of centromere biology and cellular function throughout different stages of cell division and in early development, where the sites and size of the kinetochore are first established (18, 65, 66). Variation at the sequence level could influence the rates and fidelity of alpha satellite replication (23). Further, epigenetic variation in centromere protein deposition could influence array stability (33) and improve our understanding of inner kinetochore protein inheritance and maintenance over time (73). Alpha satellite RNA transcripts have been associated with centromere function (20, 59, 60), proximity of the nucleolus (14), and genome instability (102, 118). We now have the ability to precisely map these transcripts to the genome and study the association of nearby transcription factors and bound polymerases (38). The structure and function of these highly repetitive regions of our genome present a large, unexplored genomic and epigenomic landscape. We are now faced with the challenge of closing the gaps in not only our genetic maps but also our epigenetic maps of these regions. Doing so will rely on new innovations in a number of long-read technologies to ensure the comprehensive assessment of methylation (67), replication (70), open chromatin (50), spatial maps (103), and long-read transcriptional data (115) from human centromeric satellite arrays. Future studies of epigenetic regulation of alpha satellites in early development, aging, and disease are expected to lead to a new era of discovery in centromere biology and function. Ultimately, access to alpha satellite assemblies will drive new high-resolution studies of basic cellular processes and regulation at human centromeres and has the potential to improve our understanding of human disease.

## Supplementary Material

Supplemental Tables 1-2
