# Supplementary material for: Variation and Evolution of Human Centromeres: A Field Guide and Perspective
Source: Annu Rev Genet. Author manuscript; Available in PMC 2022 Oct 10. (PMC9549924; doi:10.1146/annurev-genet-071719-020519)
Supplement: Supplemental Tables 1-2 [file NIHMS1817251-supplement-Supplemental_Tables_1-2.pdf]

Supplemental Material: Annu. Rev. Genet. 2021. 55:583-602
https://doi.org/10.1146/annurev-genet-071719-020519
Variation and Evolution of Human Centromeres: A Field Guide and Perspective
Miga and Alexandrov

Supplemental Table 1. **Suprachromosomal Families (SFs) in human alpha satellites.**

<table>
<tr><th>SF</th><th colspan="2">Monomer classes</th><th>AB</th><th>Monomer length (bp)</th><th>Typical arrangement</th><th>Age group</th><th>Age (myr)</th><th>Primate</th></tr>
<tr><td>SF1+</td><td colspan="2">J1(A) & J2(B)</td><td rowspan="4">AB</td><td rowspan="5">171</td><td rowspan="3">Chromosome-specific HORs</td><td rowspan="3">New</td><td rowspan="3">7-16</td><td rowspan="3">Human, Chimpanzee, Gorilla</td></tr>
<tr><td>SF2+</td><td colspan="2">D1(B) & D2(A)</td></tr>
<tr><td>SF3</td><td colspan="2">W1(B) & W2(B) & W3(B) & W4(A) & W5(A)</td></tr>
<tr><td>SF5</td><td colspan="2">R1(B) & R2(A) Blue</td><td>Irregular</td><td rowspan="4">Old</td><td>~16</td><td>Orangutan</td></tr>
<tr><td rowspan="8">SF4+</td><td rowspan="8">M1+</td><td>SF4 (proper): Yellow (Ga)</td><td rowspan="3">A</td><td rowspan="3">Monomeric</td><td>16-23</td><td>Gibbon</td></tr>
<tr><td>SF6 Brown (Ha)</td><td>171+172</td><td>~23</td><td>Proconsul?</td></tr>
<tr><td>SF7 Bright green (Ka)</td><td rowspan="6">172</td><td>23-26</td><td>OWM</td></tr>
<tr><td>SF8 Olive-green (Oa & Na)</td><td>A/ preA</td><td>Dimeric</td><td rowspan="5">Ancient</td><td>23-26</td><td>Extinct</td></tr>
<tr><td>SF9 Red (Ca)</td><td rowspan="4">preA</td><td rowspan="4">Monomeric</td><td>~26</td><td>Extinct</td></tr>
<tr><td>SF10 Orange (Ba)</td><td>26-40</td><td>Extinct</td></tr>
<tr><td>SF11 Lilac (Ja)</td><td>26-40</td><td>NWM</td></tr>
<tr><td>SF12 Grey (Aa)</td><td>40-58</td><td>Extinct</td></tr>
</table>

The table summarizes the data as presented in Figure S2, Supplementary tables and Text S1 in Shepelev et al. (7). Designations in columns are described below.

"SFs" (Suprachromosomal Families) in AS are designated as follows. SF1+ and SF2+ stand for SF1 and SF01, and SF2 and SF02, respectively (see the text). SF4+ stands for SF4 proper and all the older SFs including SF6, SF7 and the ancient SFs.

"Monomer classes" are SF-specific groupings (1, 2) which reflect monomer composition of distinct ancestral repeats which gave rise to each SF (e.g. a J1J2 dimer for SF1, a D1D2 dimer for SF2 and W1-W5 pentamer for SF3). M1+ is an umbrella group which unites M1 (Ga) monomers and the monomers of all the older dead layers shown as Ba, Ca, Ja, etc. For monomer classes which belong to major alpha satellite types A (permissive towards pJalpha sites) and B (permissive towards CENP-B sites), the types are indicated in parentheses.

Column "AB" indicates the monomer type composition of a given SF. The type can be determined directly by using an AB classification module of the PERCON program (8) which determines the alternative nucleotide configurations of the AB box (positions 35-51) in each monomer. Also, for long arrays, the type composition can be known tentatively by frequent (in homogeneous alpha satellite) or occasional (in divergent alpha satellite) occurrence of pJalpha (CTAYGGTGRAAAAGGAA) and CENP-B (YTTCGTTGGAARCGGGA) sites using short match option in UCSC Browser (4, 5). The "A" arrays have only pJalpha sites, the "AB"arrays have both or only CENP-B sites (as actual pJalpha sites are lost in many HORs but their monomers still remain the A-type, see in the text). "Pre-A" stands for SFs which have neither pJalpha, nor CENP-B sites.

"Monomer length" of 171 bp (characteristic of the ape live centromeres) or 172 bp (characteristic of monkeys) reflects the deletion in position 23 of the 172 bp monomer which first occurs as polymorphism in one fourth of the monomers in the brown layer (Ha) and in all younger monomers (7).

"Typical arrangement" shows the ancestral arrangement for each SF. Thus, the alteration of R1 and R2 monomers in SF5 is indicated as irregular (6), although some segments of the irregular ancestral sequence have been amplified yielding SF5 HORs. Same stands for SF4 and SF6 which both have homogeneous HORs in addition to divergent monomeric layers (see Supplementary Table 2).

"Age group" is just an arbitrary grading based on the data in the next two columns.

"Age" gives an approximate age of each SF based on the data in Shepelev et al. (7) (also see the text in this paper).

"Primate" names the primate taxon in which the respective SF was presumably an active centromere. Based on data in Shepelev (7). "OWM" and "NWM" stand for Old World Monkeys and New World Monkeys, respectively. "Proconsul" stands for the extinct primate taxon which is reliably placed between apes and the OWM (3), see discussion in Shepelev et al. (7).

Supplemental Table 2. The list of alpha satellite homogeneous HORs in hg38 human genome assembly. The Table shows the non-redundant list of alpha satellite HOR reference models composed as described in Shepelev et al. (8), with the exception of a few short models de-validated in Uralsky et al. (9). The close-knit sub-SF groups are shown by color.

#	Chromosome	RM name or clone	RM size (bp)	Number of monomers in a HOR	State	HOR name*
**1**	chr1	GJ211836.1	198 076	11	pseudo	S3C1pH2-B
**2**	chr1	GJ211837.1	278 512	11	pseudo	S3C1pH2-A
**3**	chr1	GJ211855.1	63 597	9	pseudo	S3C1qH2-D
**4**	chr1	GJ211857.1	83 495	9	pseudo	S3C1qH2-C
**5**	chr1,5,19**	GJ212202.1	2 282 185	6	live	S1C1/5/19H1L (D1Z7/D5Z2/D19Z3)
**6**	chr2	GJ211860.1	1 902 412	4	live	S2C2H1L (D2Z1)
**7**	chr2	AC026273.7	-	10	pseudo	S2C2qH2-A
**8**	chr2	AC025223.6	-	10	pseudo	S2C2qH2-B
**9**	chr3	GJ211866.1	461 128	10	pseudo	S01C3H2
**10**	chr3	GJ211871.1	2 102 155	17	live	S01/1C3H1L (D3Z1)
**11**	chr4	GJ211881.1	2 031 890	19	live	S2C4H1L (D4Z1)
**12**	chr4	AC027271.7	-	13	pseudo	S5C4H2
**13**	chr5	GJ211882.X***	83 162	16	pseudo	S5C5/19pH5
**14**	chr5	GJ211883.1	227 563	15	pseudo	S5C5pH6
**15**	chr5	GJ211884.1	264 463	32	pseudo	S5C5/19pH7-A
**16**	chr5	GJ211886.1	46 345	24	pseudo	S5C5/19pH7-B
**17**	chr5	GJ211887.1	142 630	16	pseudo	S1C5pH2
**18**	chr5,19**	GJ211904.2	53 672	17	pseudo	S5C5/19H4-A1
**19**	chr5,19**	GJ211906.2	338 504	13	pseudo	S5C5/19H4-B
**20**	chr6	GJ211907.1	1 276 046	18	live	S01C6H1L (D6Z1)
**21**	chr7	GJ211908.1	2 658 581	6	live	S1C7H1L (D7Z1)
**22**	chr7	GJ212194.1	150 232	16	pseudo	S5C7H2 (D7Z2)
**23**	chr8	GJ211909.1	1 843 521	11	live	S2C8H1L (D8Z2)
**24**	chr9	GJ211929.1	2 128 923	7	live	S2C9H1L (D9Z4)
**25**	chr10	GJ211930.1	249 218	18	pseudo	S1C10H2
**26**	chr10	GJ211932.1	1 561 440	8	live	S1C10H1L (D10Z1)
**27**	chr10	GJ211933.1	48 180	14	pseudo	S1C10H1-B
**28**	chr10	GJ211936.1	47 701	8	pseudo	S1C10H1-C
**29**	chr11	GJ211938.1	11 969	16	pseudo	S5C11H4
**30**	chr11	GJ211943.1	3 251 982	5	live	S3C11H1L (D11Z1)
**31**	chr11	GJ211948.1	82 575	12	pseudo	S3C11H2
**32**	chr12	GJ211949.1	47 204	18	pseudo	S1C12H2
**33**	chr12	GJ211954.1	2 349 957	8	live	S1C12H1L (D12Z3)

**34**	chr13,14,21,22**	GJ211955.2	22 537	33	pseudo	S4/6C13/14/21/22H8
**35**	chr13,14,21,22**	GJ211961.2	88 022	23	pseudo	S4/6C13/14/21H1
**36**	chr13,14,21,22**	GJ211962.2	54 133	20	pseudo	S6C22H2-B
**37**	chr13,14,21,22**	GJ211963.2***	63 535	20	pseudo	S6C22H2-A
**38**	chr13,14,21,22**	GJ211965.2	20 670	22	pseudo	S5C13/14/21H2
**39**	chr13,14,21,22**	GJ211967.2	6 670	16	pseudo	S4C22H3
**40**	chr13,14,21,22**	GJ211968.2	3 245	8	pseudo	S4C13/14/21/22H9
**41**	chr13,14,21,22**	GJ211969.2	22 561	17	pseudo	S4C13/14/21/22H4
**42**	chr13,14,21,22**	GJ211972.2	1 134 211	8	live	S2C14/22H1L (D14Z9/D22Z?)
**43**	chr13,21	FP565424.8	1 -	13	pseudo	S2C13/21-B
**44**	chr13,14,21,22**	GJ211991.2	632 586	11	live	S2C13/21H1L (D13Z1/D21Z1)
**45**	chr15	GJ212036.1	415 278	25	pseudo	S4C15H3
**46**	chr15	GJ212042.1	855 957	20	pseudo	S4C15H2
**47**	chr15	GJ212045.1	1 370 146	11	live	S2C15H1L (D15Z3)
**48**	chr16	GJ212046.1	23 302	12	pseudo	S2C16pH2-B
**49**	chr16	GJ212051.1	1 928 003	10	live	S1C16H1L (D16Z2)
**50**	chr17	GJ212053.1	381 239	14	epi	S3C17H1-B (D17Z1-B)
**51**	chr17	GJ212054.1***	3 371 615	16	live	S3C17H1L (D17Z1)
**52**	chr17	GJ212055.1	49 431	14	pseudo	S3C17H1-C (D17Z1-C)
**53**	chr18	GJ212060.1	319 478	10	pseudo	S2C18pH2-A
**54**	chr18	GJ212062.1	4 763 584	12	live	S2C18H1L (D18Z1)
**55**	chr18	GJ212066.1	93 042	10	pseudo	S2C18qH2-B
**56**	chr18	GJ212067.1	39 636	11	pseudo	S2C18qH2-D
**57**	chr18	GJ212071.1***	21 409	11	pseudo	S2C18qH2-E
**58**	chr20	GJ212091.1	150 723	8	pseudo	S2C20H2
**59**	chr20	GJ212093.1	1 886 394	16	live	S2C20H1L (D20Z2)
**60**	chr20	GJ212095.1	47 956	11	pseudo	S02C20H3
**61**	chr20	GJ212105.1	80 766	11	pseudo	S4C20H4
**62**	chr20	GJ212117.1	120 944	8	pseudo	S5C20H6
**63**	chrX	GJ212192.1	3 806 963	12	live	S3CXH1L (DXZ1)
**64**	chrY	GJ212193.1	227 095	34	live	S4CYH1L (DYZ3)

*New names were assigned to HORs in hg38 alpha satellite reference models following the rules proposed in Uralsky et al. (9). The parts of the HOR names which reflect the chromosomal locations were adjusted for few HORs to reflect locations observed in CHM13 T2T genome assembly (2).

** Only one representative member of a group of identical reference models is listed. For a complete list see Supplementary Table S1 in Shepelev et al. (8).

*** Corrected versions of these reference models were provided by K. Miga as described previously (8).

REFERENCES

1. Alexandrov I, Kazakov A, Tumeneva I, Shepelev V, Yurov Y. 2001. Alpha-satellite DNA of primates: old and new families. *Chromosoma*. 110(4):253–66
2. Altemose N. 2021. Complete genomic and epigenetic maps of human centromeres. *bioRxiv*
3. Goodman M, Porter CA, Czelusniak J, Page SL, Schneider H, et al. 1998. Toward a phylogenetic classification of Primates based on DNA evidence complemented by fossil evidence. *Mol. Phylogenet. Evol.* 9(3):585–98
4. Kent WJ, Sugnet CW, Furey TS, Roskin KM, Pringle TH, et al. 2002. The human genome browser at UCSC. *Genome Res.* 12(6):996–1006
5. Raney BJ, Dreszer TR, Barber GP, Clawson H, Fujita PA, et al. 2014. Track data hubs enable visualization of user-defined genome-wide annotations on the UCSC Genome Browser. *Bioinformatics*. 30(7):1003–5
6. Romanova LY, Deriagin GV, Mashkova TD, Tumeneva IG, Mushegian AR, et al. 1996. Evidence for selection in evolution of alpha satellite DNA: the central role of CENP-B/pJ alpha binding region. *J. Mol. Biol.* 261(3):334–40
7. Shepelev VA, Alexandrov AA, Yurov YB, Alexandrov IA. 2009. The evolutionary origin of man can be traced in the layers of defunct ancestral alpha satellites flanking the active centromeres of human chromosomes. *PLoS Genet.* 5(9):e1000641
8. Shepelev VA, Uralsky LI, Alexandrov AA, Yurov YB, Rogaev EI, Alexandrov IA. 2015. Annotation of suprachromosomal families reveals uncommon types of alpha satellite organization in pericentromeric regions of hg38 human genome assembly. *Genom Data*. 5:139–46
9. Uralsky LI, Shepelev VA, Alexandrov AA, Yurov YB, Rogaev EI, Alexandrov IA. 2019. Classification and monomer-by-monomer annotation dataset of suprachromosomal family 1 alpha satellite higher-order repeats in hg38 human genome assembly. *Data Brief.* 24:103708
